# Psychological, behavioural, and physical aspects of caregiver strain in autism-caregivers: a cohort study

**DOI:** 10.1016/j.eclinm.2023.102211

**Published:** 2023-09-20

**Authors:** Eva B. Warreman, Susan E. Lloyd, Laura A. Nooteboom, Pieter J.M. Leenen, Mary Beth Terry, Hans W. Hoek, Elisabeth F.C. van Rossum, Robert R.J.M. Vermeiren, Wietske A. Ester

**Affiliations:** a Department of Child and Adolescent Psychiatry, LUMC Curium, Leiden University Medical Centre, Endegeesterstraatweg 27, Oegstgeest 2342 AK, the Netherlands; b Department of Epidemiology, Mailman School of Public Health, Columbia University, 722 West 168th St., New York, United States; c Department of Immunology, Erasmus University Medical Centre, PO Box 2040, Rotterdam 3000 CA, the Netherlands; d Department of Psychiatry, University Medical Centre Groningen, Hanzeplein 1, Groningen 9713 GZ, the Netherlands; e Parnassia Psychiatric Institute, Youz, Kiwistraat 43, The Hague 2552 DH, the Netherlands; f Department of Internal Medicine, Erasmus University Medical Centre, PO Box 2040, Rotterdam 3000 CA, the Netherlands; g Sarr Autism Rotterdam, Youz Child and Adolescent Psychiatry, Dynamostraat 18, Rotterdam 3083 AK, the Netherlands

**Keywords:** Autism, Caregiving, Health, Strain

## Abstract

**Background:**

People who give care to autistic individuals (autism-caregivers) experience higher levels of caregiver strain than people who provide care for individuals with other chronic conditions (non-autism-caregivers). This places them at higher risk for psychological, behavioural and physical health concerns. The aim of this study is to delineate psychological, behavioural, and physical aspects of caregiver strain in autism-caregivers compared to non-autism-caregivers.

**Methods:**

We included 3354 adult caregivers from the general population in the Netherlands participating in the second assessment (January, 1, 2014–December, 31, 2017) of the Lifelines Cohort. In this cohort study, using multivariable regression adjusted for age, sex, and socioeconomic status, we analysed psychological (anxiety and depression based on a Mini International Neuropsychiatric Interview, and self-reported stress and perceived health), behavioural (questionnaire-assessed physical activity, alcohol use, and smoking), and physical aspects (body mass index, waist circumference, and leukocyte-counts) of caregiver strain in autism-caregivers (n = 722) compared with non-autism-caregivers (n = 2632).

**Findings:**

Autism-caregivers reported more stress (OR 3.61, 95% CI 2.60–4.99). Both anxiety (OR 1.85, 95% CI 1.37–2.49) and depressive disorders (OR 1.83, 95% CI 1.17–2.86) were more common in autism-caregivers than in non-autism-caregivers. Perceived health, physical activity, alcohol use, and smoking were not different between autism- and non-autism-caregivers. In autism-caregivers, lymphocyte- and monocyte-counts were lower than in non-autism-caregivers.

**Interpretation:**

In this large cohort, autism-caregivers had worse psychological health than non-autism-caregivers. Moreover, autism-caregiving might be associated with an altered immune balance. These findings underline the higher caregiver strain in autism-caregivers compared to other caregivers. This calls for increased support to autism-caregivers.

**Funding:**

Lifelines has been funded by the Dutch government.

**Research in context**

**Evidence before this study**

A PubMed search including meta-analyses and (systematic) reviews from January 1, 2000, to May 31, 2023, using the search terms “caregiver/caregiving”, “autism/autistic”, “strain/health/distress/burden” mainly resulted in studies regarding parents of autistic children. Parents of autistic children seem to experience more stress and worse quality of life than other parents. Previous studies also found that there is a bidirectional relationship between strain in autism-caregivers and the child’s internalizing and externalizing behaviours.

**Added value of this study**

This study offers an integrated investigation of psychological, behavioural, and biological aspects of caregiver strain in a large sample of autism-caregivers, compared to other types of caregivers (non-autism-caregivers). The impact of autism-caregiving on different aspects of caregiver strain was investigated using both biomarkers and self-reported data. This integrated approach and the inclusion of both parental and non-parental autism-caregivers is a valuable addition to previous research, since previous studies assessed caregiver strain in autism-caregivers more fragmentarily and mainly in parental autism-caregivers.

**Implications of all the available evidence**

Adding up the results of both previous research and the current study, it is evident that autism-caregivers are specifically at increased risk for adverse psychological caregiver strain. Moreover, the investigated psychological aspects of caregiver strain are not different between parental and non-parental autism-caregivers. In addition, autism-caregiving is related to an altered lymphocyte balance. Taken together, the available evidence calls for increased support to autism-caregivers. Therefore, future research focusing on implementation of interventions to prevent and reduce caregiver strain in both parental and non-parental autism-caregivers would be valuable.

## Introduction

People who are caregiver for someone with a chronic condition can experience different objective and subjective aspects of caregiver strain, such as negative effects on finances, work, relationships, and psychological, behavioural and physical health.^1^

Caregiver burden is higher in people who provide care for autistic individuals (autism-caregivers) than in people who provide care for individuals with other chronic conditions (non-autism-caregivers).^2^ This might lead to a greater risk for various types of health problems among autism-caregivers compared to non-autism-caregivers, and even for higher mortality rates in some cases.^3^^,^^4^ Previous studies have identified several psychological, behavioural, and physical aspects of caregiver strain in general, but not specifically for autism-caregivers.^5^ To improve autism-caregivers’ health and well-being, more insight into psychological, behavioural, and physical aspects of their health is needed.^4^^,^^6^

Stress is an important psychological aspect of caregiver strain. The observed greater mortality risk for autism-caregivers might be related to higher stress levels, as autism-caregivers experience on average more caregiver stress than non-autism-caregivers.^3^^,^^7^ Moreover, there is a relationship, which can intensify through bidirectional feedback, between stress levels in parents of autistic children (parental autism-caregivers) and internalizing and externalizing behaviours in these children.^8^ However, we hypothesise that these increased stress-levels are not only present in parental autism-caregivers, but also in adults who are caregiver for another autistic family member or friend (non-parental autism-caregivers), because being a lifelong non-parental autism-caregiver might also be more stressful than being a non-parental non-autism-caregiver. Another relevant psychological aspect in caregiver strain is self-perceived health, as caregivers have reported worse health than non-caregivers.^9^ Moreover, anxiety and depression are highly prevalent in parental autism-caregivers, which seems to be mediated by parenting stress, and thus important to examine in non-parental autism-caregivers as well.^10^^,^^11^

Behavioural aspects like smoking, alcohol use, and physical activity are important when assessing caregiver strain, because these are lifestyle risk factors for non-communicable diseases. Moreover, smoking and alcohol consumption can be part of a coping mechanism for caregiver stress. Parents of autistic children seem to be more frequently users of alcohol and tobacco compared to the general population.^12^ To our knowledge, smoking and alcohol use have only been investigated in parental autism-caregivers, and not in both parental and non-parental autism-caregivers.^12^ Furthermore, the prevalence of smoking, alcohol use, and physical activity in (both parental and non-parental) autism-caregivers compared to non-autism-caregivers is still unknown.

Lastly, physical aspects of caregiver strain can be assessed with physiological markers related to caregiver stress, including immune components responding to hypothalamic-pituitary-adrenal (HPA) axis activity, such as leukocyte- and -subtype counts. However, with regard to HPA axis activity in autism-caregivers, previous research focused on salivary cortisol, which revealed altered cortisol levels in parental autism-caregivers compared to parental non-autism-caregivers.^13^ In addition, body mass index (BMI) and waist circumference should be taken into account, because of their association with for example cardiovascular and cancer risk.^14^ Only BMI has been previously described in parental autism-caregivers, but their BMI was not different than in parental non-autism-caregivers.^15^

In summary, a better understanding of autism-caregivers strain is needed, because of our first hypothesis regarding increased health risks in autism-caregivers compared to non-autism-caregivers. Therefore, the first aim of this study is to compare the above-mentioned psychological, behavioural, and physical aspects of caregiver strain in autism-caregivers and non-autism-caregivers. Moreover, we hypothesise that autism-caregiving is associated with higher caregiver strain than non-autism-caregiving, irrespective of being a parent. However, to our knowledge, caregiver strain has not previously been compared between parental and non-parental autism-caregivers. Therefore, to investigate this second hypothesis, we also aim to compare the above-mentioned psychological, behavioural, and physical aspects of caregiver strain between parental autism-caregivers and non-parental autism-caregivers, and between parental autism-caregivers and parental non-autism-caregivers.

## Methods

### Study population

**Fig. 1:**
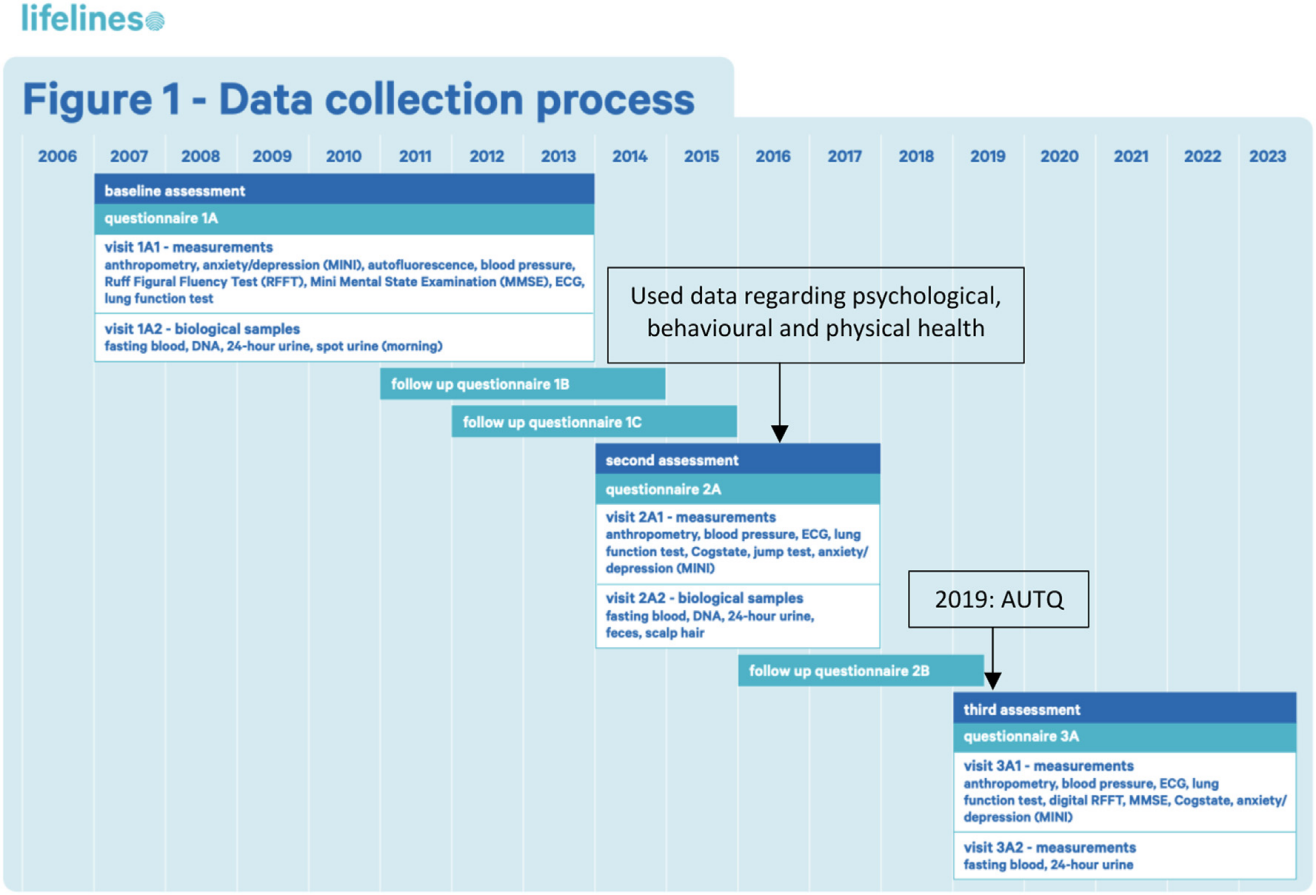
**Data collection process∗.** AUTQ = autism and caregiver questionnaire. ∗Downloaded from: https://www.lifelines.nl/researcher/data-and-biobank.

**Fig. 2:**
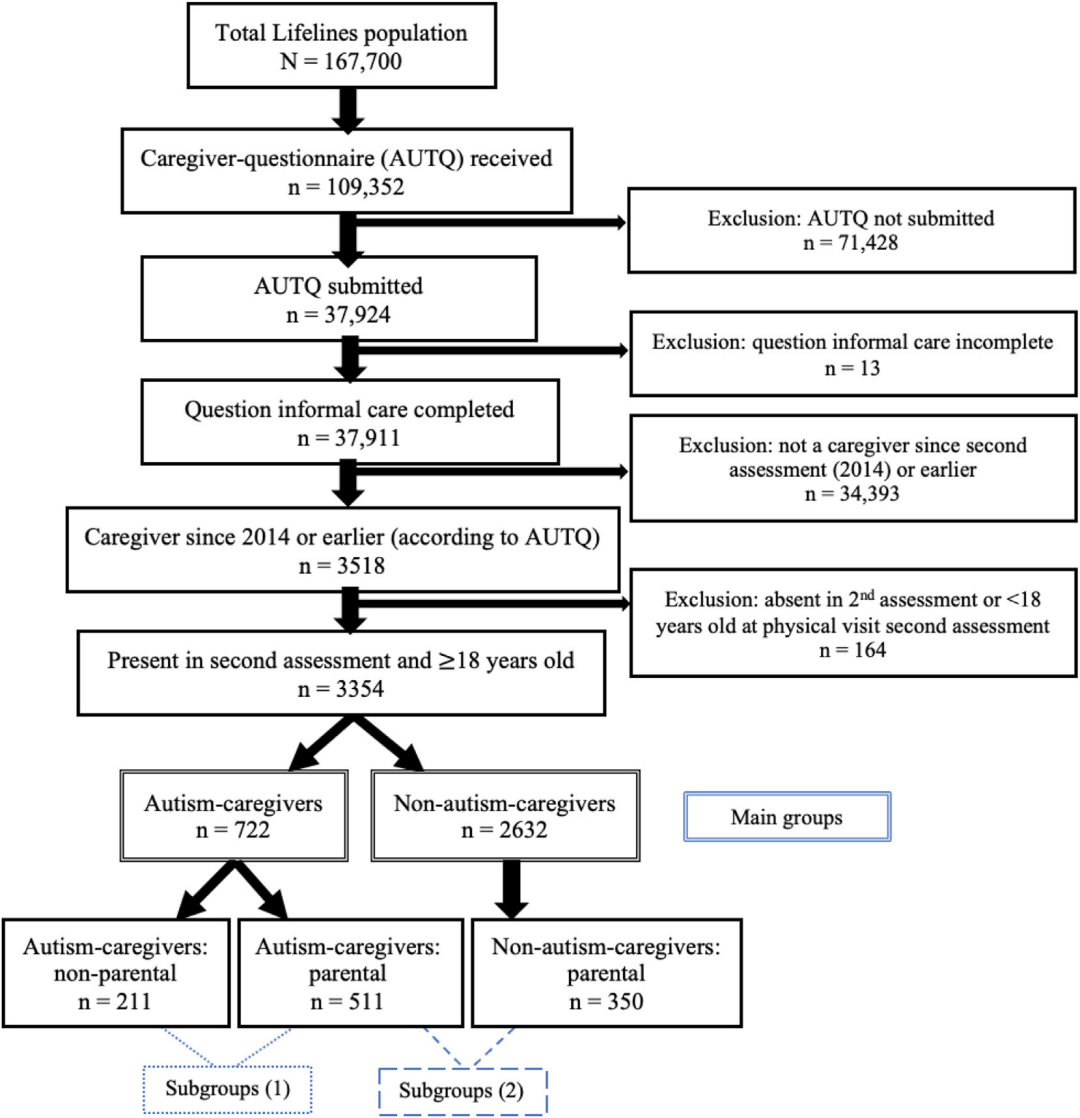
**Flow diagram of study population.** AUTQ = autism and caregiver questionnaire.

All data used in this study were extracted from the Lifelines Cohort database. The Lifelines protocol was approved by the UMCG Medical ethical committee under number 2007/152. Lifelines is a multi-disciplinary prospective population-based cohort study examining the health and health-related behaviours of 167,729 persons living in the North of the Netherlands in a unique three-generation design. These participants were included through general practitioners, which resulted in a general population sample. Lifelines employs a broad range of investigative procedures in assessing the biomedical, sociodemographic, behavioural, physical, and psychological factors that contribute to the health and disease of the general population, with a special focus on multi-morbidity and complex genetics.^16^ Baseline assessment was performed from 2007 until 2013, and the second assessment took place between January, 1st, 2014 and December, 31st, 2017 (Fig. 1). In 2019, 109,352 participants received an autism and caregiver questionnaire (AUTQ). In order to evaluate potential selection bias, we executed a non-response analysis to map characteristics of the 71,428 Lifelines participants that did receive the AUTQ, but did not submit this questionnaire (and could therefore not be included in our study, see Fig. 2). This non-response analysis showed that these 71,428 non-eligible Lifelines participants were younger in age and consisted of more men compared to the 37,924 participants who did submit this AUTQ questionnaire.

In this cohort study, we included 3354 participants who reported being an informal unpaid caregiver to someone close prior to 2015 (since the second Lifelines assessment started in 2014), and who were age 18 years or older during the physical visit of the second Lifelines assessment (Fig. 2). In this way, the caregiving began prior to the outcome measurement of caregiver strain. Of the 3354 included caregivers (Fig. 2), 722 were autism-caregiver and 2632 were non-autism-caregiver. These autism-caregivers consisted of 511 parental autism-caregivers (71.9%). In the group of non-autism-caregivers, there were 350 parental non-autism-caregivers (13.3%). Parental caregivers were adults who gave care to their son or daughter (in law). Non-parental caregivers were adults who gave care to their partner, mother (in law), father (in law), brother, sister, friend, acquaintance, or neighbour.

### Measures

#### Caregiving

The AUTQ assessed whether or not participants were informal caregivers, and if so, for how long they have been a caregiver, what type of condition their care-receivers had and what type of relative/acquaintance their care-receiver was. Informal caregiving was defined as giving unpaid care to someone close with long-term limitations and/or health problems. ‘Someone close’ was described as a partner, a relative (including a child), a friend, or someone else close to you. Volunteer work was not included in our definition of caregiving.

#### Psychological aspects of caregiver strain

##### Chronic stress

Self-reported chronic stress was investigated with the Long-term Difficulties Inventory (LDI).^17^ LDI sum-scores were calculated to compare stress levels between groups. The LDI sum-scores ranged from 0 to 19 and were divided in four categories (0, 1–2, 3–4, ≥5) for unordered polytomous analyses.

##### Perceived health

Perceived health was assessed with a question from the RAND-36-Item Health Survey about general health, in which participants answered on a 5-point Likert scale how they would rate their health generally speaking: poor, mediocre, good, very good, excellent.^18^

#### Anxiety and depressive disorder

The presence of an anxiety or depressive disorder was determined in a face-to-face Mini International Neuropsychiatric Interview (MINI), based on the DSM-IV-TR.^19^ Anxiety was defined as the presence of any current anxiety disorder: panic disorder, agoraphobia, social phobia, or generalized anxiety disorder. Depression included the presence of any current depressive disorder: major depressive disorder or dysthymia.

#### Behavioural aspects of caregiver strain

##### Physical activity

Physical activity was measured using the following question from the Short Questionnaire to Assess Health-enhancing physical activity (SQUASH): ‘Adding everything up, on how many days per week on average are you involved in cycling, doing odd jobs, gardening, sport, or other strenuous activities for at least 30 min?‘.^20^ This variable was analysed binary, using a cut-off point of ≥5 days a week of performing at least 30 min of physical activity (≥150 min/week).

##### Smoking

Participants were categorized as a smoker if they answered the following question with ‘yes’: “Do you smoke now or have you smoked in the past month?”.

##### Alcohol use

Alcohol use was assessed with a question from the Flower Food Frequency questionnaire: ‘During the past month, how many glasses of alcoholic drinks did you drink per day on average?‘.^21^ An average alcohol intake of more than two glasses per day (heavy drinking) was taken as cut-off point in analyses.

#### Physical aspects of caregiver strain

##### Body mass index

Height and weight (for calculation of BMI) were measured by trained Lifelines personnel during a physical visit of the second assessment. Moreover, overweight (BMI 25–29.99 kg/m^2^) and obesity (BMI ≥ 30 kg/m^2^) were investigated.

##### Waist circumference

Waist circumference was also measured by trained Lifelines personnel during a physical visit of the second assessment. Waist circumference was analysed as a continuous variable. Besides, a waist circumference above the threshold for metabolic syndrome (≥88 cm in women, ≥102 cm in men) was used as a cut-off point in analyses.^22^

##### Leukocytes and subtypes

Blood samples were drawn during a physical visit of the second assessment. Leukocyte- and -subtype counts were analysed as measures of (low-grade) inflammation, regulated by HPA axis activity. The investigated leukocyte-subtypes included neutrophils, lymphocytes, monocytes, eosinophils, and basophils. Lastly, the neutrophil-to-lymphocyte ratio was analysed by dividing the neutrophil count to the lymphocyte count.

#### Covariates

##### AQ-10

The short version of the Autism Spectrum Quotient (AQ-10) was used to quantify the degree of autistic traits in caregivers.^23^

#### Employment

Self-reported employment status and educational attainment were used in analyses as measures of socioeconomic status. Participants were categorized as being employed if they currently had paid work for one or more hours per week.

##### Education

Educational attainment was categorised in four groups: low (no education, primary, lower or preparatory vocational education, or lower general secondary education), middle (intermediate vocational education or apprenticeship, higher general secondary education, or pre-university secondary education), high (higher vocational education or university), or other.

### Statistical analysis

IBM SPSS Statistics version 25 was used for all data analyses. We executed descriptive statistics to compare age, sex, educational attainment, employment, ethnicity, number of people in the household, the type of person they were caregiver for, and the AQ-10 sum-score between autism-caregivers and non-autism-caregivers, between parental autism-caregivers and non-parental autism-caregivers, and between parental autism-caregivers and parental non-autism-caregivers. For these comparisons of demographics, we used Student *t* tests or Mann–Whitney U tests for continuous variables, and Chi-square tests for categorical variables (Table 1). Next, we conducted separate multivariable multinomial, logistic, or quantile regression models to examine the association between caregiver type (autism-caregiver versus non-autism caregiver) and each of the above-mentioned psychological, behavioural, and physical aspects of caregiver-strain as outcomes (Table 2). Stress and perceived health were analysed with unordered multinomial regression analyses. Binary outcomes, including anxiety, depression, physical activity, smoking, alcohol use, overweight, obesity, and waist circumference above the threshold were analysed using logistic regression analyses. BMI, waist circumference, leukocytes, and leukocyte-subtypes were analysed with quantile regression. Because of missing data in the covariates of employment and educational attainment (see all missing data in Supplementary Table S1), we performed step-by-step analyses: model 1 adjusted only for age and sex; model 2 adjusted for age, sex, and employment; model 3 adjusted for age, sex, employment, and educational attainment. Lastly, we performed the same multivariable regression analyses in the subgroups, with (1) being a parental or non-parental autism-caregiver as independent variable, and (2) being a parental autism-caregiver or parental non-autism-caregiver as independent variable (Table 3). Since the results remained similar between model 1, 2, and 3 in the main analyses (Table 2), the parental subgroup analyses were performed with model 3: adjusted for age, sex, employment, and educational attainment. In these parental subgroup-analyses, the lowest two categories of perceived health (poor and mediocre) were combined due to small response in the “poor” category.

**Table 1:** Basic characteristics in autism-caregivers compared to non-autism-caregivers.

	Autism-caregivers n = 722	Non-autism-caregivers n = 2632	p-value^a^	Autism-caregivers: parental n = 511	Autism-caregivers: non-parental n = 211	p-value^a^	Non-autism-caregivers: parental n = 350	p-value^b^
Age (mean, SD)^c^	50.8 (9.1)	53.8 (9.0)	<0.001	50.0 (8.2)	52.6 (10.8)	<0.001	53.6 (9.4)	<0.001
Female sex (N, %)	541 (74.9)	1794 (68.2)	<0.001	395 (77.3)	146 (69.2)	0.022	247 (70.6)	0.026
Educational attainment								
Low (N, %)	104 (14.4)	577 (21.9)	<0.001	73 (14.3)	31 (14.7)	0.987	84 (24.0)	0.003
Medium (N, %)	260 (36.0)	879 (33.4)		188 (36.8)	72 (34.1)		112 (32.0)	
High (N, %)	211 (29.2)	735 (27.9)		143 (28.0)	68 (32.2)		111 (31.7)	
Employment (N, %)	491 (68.0)	1777 (67.5)	0.098	356 (69.7)	135 (64.0)	0.063	223 (63.7)	<0.001
Ethnicity^d^								
Eastern or Western European (N, %)	659 (91.3)	2463 (93.6)	0.038	457 (89.4)	202 (95.7)	0.236	326 (93.1)	0.057
Mediterranean or Arabic (N, %)	<10 (<1.4)	<10 (<0.4)		<10 (<2.0)	<10 (<4.7)		<10 (<2.9)	
Asian (N, %)	<10 (<1.4)	<10 (<0.4)		<10 (<2.0)	<10 (<4.7)		<10 (<2.9)	
Other (N, %)	10 (1.4)	12 (0.5)		<10 (<2.0)	<10 (<4.7)		<10 (<2.9)	
Number of people in household (median, IQR)^e^	3 (2–4)	2 (2–3)	0.000	4 (2–4)	2 (2–3)	<0.001	3 (2–4)	<0.001
Caregiver of …								
Partner (N, %)	69 (9.6)	424 (16.1)	<0.001	31 (6.1)	38 (18.0)	<0.001	16 (4.6)	0.343
Mother and/or father (in law) (N, %)	156 (21.6)	1607 (61.1)	<0.001	99 (19.4)	57 (27.0)	0.023	83 (23.7)	0.125
Son and/or daughter (in law) (N, %)	511 (70.8)	350 (13.3)	<0.001	511 (100)	<10 (<4.7)	<0.001	350 (100)	–
Brother and/or sister (N, %)	84 (11.6)	207 (7.9)	0.001	11 (2.2)	73 (34.6)	<0.001	<10 (<2.9)	0.650
Friend or acquaintance (N, %)	38 (5.3)	162 (6.2)	0.370	11 (2.2)	27 (12.8)	<0.001	<10 (<2.9)	0.878
Neighbour (N, %)	10 (1.4)	98 (3.7)	0.002	<10 (<2.0)	<10 (<4.7)	0.451	<10 (<2.9)	0.744
Other (N, %)	81 (11.2)	232 (8.8)	0.049	12 (2.3)	69 (32.7)	<0.001	12 (3.4)	0.3444
AQ-10 sum score (median, IQR)	2 (1–4)	2 (1–3)	0.676	2 (1–4)	2 (1–3)	0.134	2 (1–3)	0.082

a Univariable analyses in which continuous variables were analysed with Student *t* tests or Mann–Whitney U tests, and categorical variables with Chi-square tests.

b Parental non-autism-caregivers (n = 350) compared to parental autism-caregivers (n = 511) using univariable analyses in which continuous variables were analysed with Student *t* tests or Mann–Whitney U tests, and categorical variables with Chi-square tests.

c SD = standard deviation.

d Assessed with the following multiple-choice question: ‘Which of the following populations do you consider yourself belonging to?’

e IQR = interquartile range.

**Table 2:** Psychological, behavioural, and physical aspects of caregiver strain: autism-caregivers versus non-autism-caregivers.

	Autism-caregivers n = 722	Non-autism-caregivers n = 2632	Being an autism-caregiver as independent variable, adjusted for age and sex (model 1)	Being an autism-caregiver as independent variable, adjusted for age, sex, and employment (model 2)	Being an autism-caregiver as independent variable, adjusted for age, sex, employment, and educational attainment (model 3)
**Psychological**					
Stress^a^					
0 (N, %)	91 (12.6)	660 (25.1)	Reference	Reference	Reference
1–2 (N, %)	232 (32.1)	970 (36.9)	OR 1.60 (95% CI 1.22–2.08), p < 0.001	OR 1.62 (95% CI 1.24–2.12), p < 0.001	OR 1.58 (95% CI 1.18–2.11), p = 0.002
3–4 (N, %)	157 (21.7)	531 (20.2)	OR 1.86 (95% CI 1.39–2.49), p < 0.001	OR 1.88 (95% CI 1.41–2.53), p < 0.001	OR 1.91 (95% CI 1.40–2.62), p < 0.001
≥5 (N, %)	169 (23.4)	291 (11.1)	OR 3.50 (95% CI 2.59–4.74), p < 0.001	OR 3.54 (95% CI 2.61–4.80), p < 0.001	OR 3.61 (95% CI 2.60–4.99), p < 0.001
**Perceived health**^b^					
Poor (N, %)	<10 (1.4)	<10 (0.4)	Reference	Reference	Reference
Mediocre (N, %)	102 (14.1)	257 (9.8)	OR 0.56 (95% CI 0.19–1.64), p = 0.294	OR 0.58 (95% CI 0.20–1.72), p = 0.324	OR 0.59 (95% CI 0.18–1.96), p = 0.390
Good (N, %)	346 (47.9)	1356 (51.5)	OR 0.38 (95% CI 0.13–1.08), p = 0.069	OR 0.39 (95% CI 0.14–1.14), p = 0.086	OR 0.40 (95% CI 0.12–1.29), p = 0.124
Very good (N, %)	149 (20.6)	616 (23.4)	OR 0.37 (95% CI 0.13–1.06), p = 0.063	OR 0.38 (95% CI 0.13–1.12), p = 0.079	OR 0.36 (95% CI 0.11–1.17), p = 0.089
Excellent (N, %)	48 (6.6)	218 (8.3)	OR 0.33 (0.11–0.99), p = 0.049	OR 0.34 (95% CI 0.11–1.03), p = 0.057	OR 0.32 (95% CI 0.10–1.09), p = 0.069
Anxiety disorder (N, %)	88 (12.2)	181 (6.9)	OR 1.72 (95% CI 1.30–2.27), p < 0.001	OR 1.82 (95% CI 1.35–2.44), p < 0.001	OR 1.85 (95% CI 1.37–2.49), p < 0.001
Depressive disorder (N, %)	43 (6.0)	74 (2.8)	OR 1.97 (95% CI 1.33–2.93), p < 0.001	OR 1.87 (95% CI 1.21–2.91), p = 0.005	OR 1.83 (95% CI 1.17–2.86), p = 0.008
**Behavioural**					
Physical activity ≥150 min/week) (N, %)^c^	348 (48.2)	1247 (47.4)	OR 1.17 (95% CI 0.98–1.39), p = 0.086	OR 1.17 (95% CI 0.98–1.39), p = 0.089	OR 1.12 (95% CI 0.93–1.35), p = 0.251
Smoking (currently or in past month) (N, %)	85 (11.8)	313 (11.9)	OR 0.96 (95% CI 0.74–1.25), p = 0.769	OR 0.98 (95% CI 0.75–1.30), p = 0.907	OR 0.97 (95% CI 0.73–1.31), p = 0.854
Alcohol use (>2 glasses/day) (N, %)	86 (11.9)	362 (13.8)	OR 0.91 (95% CI 0.68–1.19), p = 0.469	OR 0.94 (95% CI 0.71–1.25), p = 0.687	OR 0.99 (95% CI 0.73–1.34), p = 0.924
**Physical**					
Body mass index (median, IQR)	25.7 (23.3–29.2)	25.8 (23.5–28.7)	β 0.05 (95% CI −0.35 to 0.45), p = 0.806	β 0.11 (95% CI −0.31 to 0.53), p = 0.606	β 0.05 (95% CI −0.39 to 0.48), p = 0.832
Overweight (N, %)	265 (36.7)	1111 (42.2)	OR 0.85 (95% CI 0.71–1.01), p = 0.063	OR 0.84 (95% CI 0.70–1.00), p = 0.051	OR 0.83 (95% CI 0.68–1.00), p = 0.055
Obesity (N, %)	146 (20.2)	450 (17.1)	OR 1.18 (95% CI 0.96–1.36), p = 0.124	OR 1.24 (95% CI 0.99–1.55), p = 0.056	OR 1.26 (95% CI 0.99–1.59), p = 0.057
Waist circumference (median, IQR)	90.0 (81.0–99.0)	90.0 (82.5–99.0)	β −0.14 (95% CI −1.31 to 1.02), p = 0.812	β −0.07 (95% CI −1.30 to 1.16), p = 0.911	β 0.22 (95% CI −1.07 to 1.51), p = 0.739
≥threshold (N, %)^d^	323 (44.7)	1129 (42.9)	OR 1.08 (95% CI 0.91–1.28), p = 0.374	OR 1.10 (95% CI 0.92–1.32), p = 0.293	OR 1.10 (95% CI 0.91–1.33), p = 0.339
Leukocytes (10^E^9/L) (median, IQR)	5.80 (4.90–6.90)	5.80 (5.00–6.90)	β −0.06 (95% CI −0.21 to 0.08), p = 0.380	β −0.05 (95% CI −0.20 to 0.10), p = 0.503	β −0.08 (95% CI −0.24 to 0.08), p = 0.335
Neutrophils (10^E^9/L) (median, IQR)	3.15 (2.53–3.99)	3.07 (2.47–3.87)	β 0.02 (95% CI −0.09 to 0.13), p = 0.708	β 0.04 (95% CI −0.08 to 0.15), p = 0.555	β 0.01 (95% CI −0.12 to 0.14), p = 0.862
Lymphocytes (10^E^9/L) (median, IQR)	1.89 (1.56–2.22)	1.93 (1.60–2.36)	β −0.07 (95% CI −0.13 to −0.01), p = 0.019	β −0.07 (95% CI −0.13 to −0.01), p = 0.022	β −0.08 (95% CI −0.15 to −0.02), p = 0.016
Monocytes (10^E^9/L) (median, IQR)	0.45 (0.38–0.55)	0.47 (0.40–0.58)	β −0.02 (95% CI −0.03 to −0.001), p = 0.034	β −0.02 (95% CI −0.04 to −0.01), p = 0.010	β −0.02 (95% CI −0.04 to −0.004), p = 0.014
Eosinophils (10^E^9/L) (median, IQR)	0.16 (0.10–0.24)	0.16 (0.11–0.24)	β 0.00 (95% CI −0.01 to 0.01), p = 1.000	β 0.00 (95% CI −0.01 to 0.01), p = 1.000	β −0.00 (95% CI −0.01 to 0.01), p = 0.940
Basophils (10^E^9/L) (median, IQR)	0.04 (0.03–0.06)	0.05 (0.03–0.06)	β −0.01 (95% CI −0.01 to −0.01), p = 0.000	β −0.000 (95% CI −0.002 to 0.002), p = 1.000	β −0.000 (95% CI −0.004 to 0.004), p = 1.000
Neutrophil-to-lymphocyte ratio (median, IQR)	1.63 (1.31–2.16)	1.60 (1.25–2.05)	β −0.01 (95% CI −0.08 to 0.06), p = 0.779	β −0.004 (95% CI −0.073 to 0.066), p = 0.921	β 0.02 (95% CI −0.05 to 0.10), p = 0.593

SD = standard deviation. IQR = interquartile range. OR = odds ratio. 95% CI = 95% confidence interval.

Multinomial, logistic, or quantile regression with a psychological, behavioural, and physical aspects of caregiver strain as dependent variable and being an autism-caregiver or non-autism-caregiver as independent variable. Model 1 adjusted only for age and sex; model 2 adjusted for age, sex, and employment; model 3 adjusted for age, sex, employment, and educational attainment.

a A higher score in the Long-term Difficulties Inventory resembles more perceived stress.

b A lower score resembles worse perceived health.

c Based on the Short Questionnaire to Assess Health-enhancing physical activity.

d Waist circumference above threshold for metabolic syndrome: ≥88 cm in women, ≥102 cm in men.

**Table 3:** Subgroup analyses: Psychological, behavioural, and physical aspects of caregiver strain in (1) autism-caregivers: parental versus non-parental, and (2) parental caregivers: parental autism-caregivers versus parental non-autism-caregivers.

	Autism-caregivers: parental, n = 511	Autism-caregivers: non-parental, n = 211	(1) Being a parental or non-parental autism-caregiver as independent variable, adjusted for age, sex, employment, and educational attainment (model 3)	Non-autism-caregivers: parental, n = 350	(2) Being a parental autism-caregiver or parental non-autism-caregiver as independent variable, adjusted for age, sex, employment, and educational attainment (model 3)
**Psychological**					
Stress^a^					
0 (N, %)	61 (11.9)	30 (14.2)	Reference	95 (27.1)	Reference
1–2 (N, %)	162 (31.7)	70 (33.2)	OR 0.74 (95% CI 0.40–1.36), p = 0.328	122 (34.9)	OR 1.52 (95% CI 0.98–2.37), p = 0.063
3–4 (N, %)	104 (20.4)	53 (25.1)	OR 0.61 (95% CI 0.32–1.16), p = 0.133	66 (18.9)	OR 1.95 (95% CI 1.19–3.20), p = 0.008
≥5 (N, %)	130 (25.4)	39 (18.5)	OR 1.03 (95% CI 0.53–1.99), p = 0.938	50 (14.3)	OR 3.10 (95% CI 1.86–5.17), p < 0.001
**Perceived health**^b^					
Poor or mediocre (N, %)	77 (15.1)	31 (14.7)	Reference	40 (11.4)	Reference
Good (N, %)	243 (47.6)	103 (48.8)	OR 0.92 (95% CI 0.55–1.53), p = 0.734	188 (53.7)	OR 0.64 (95% CI 0.40–1.01), p = 0.056
Very good (N, %)	109 (21.3)	40 (19.0)	OR 1.27 (95% CI 0.69–2.34), p = 0.446	78 (22.3)	OR 0.71 (95% CI 0.42–1.20), p = 0.205
Excellent (N, %)	30 (5.9)	18 (8.5)	OR 0.88 (95% CI 0.39–1.97), p = 0.756	28 (8.0)	OR 0.60 (95% CI 0.30–1.20), p = 0.147
Anxiety disorder (N, %)	65 (12.7)	23 (10.9)	OR 1.08 (95% CI 0.62–1.88)	24 (6.9)	OR 1.87 (95% CI 1.09–3.25)
Depressive disorder (N, %)	30 (5.9)	13 (6.2)	OR 0.83 (95% CI 0.39–1.79)	12 (3.4)	OR 1.75 (95% CI 0.80–3.20)
**Behavioural**					
Physical activity ≥150 min/week) (N, %)^c^	242 (47.4)	106 (50.2)	OR 0.90 (95% CI 0.62–1.30), p = 0.560	170 (48.6)	OR 1.07 (95% CI 0.79–1.46), p = 0.653
Smoking (currently or in past month) (N, %)	69 (13.5)	16 (7.6)	OR 2.36 (95% CI 1.19–4.70)	44 (12.6)	OR 1.08 (95% CI 0.69–1.70)
Alcohol use (>2 glasses/day) (N, %)	65 (12.7)	21 (10.0)	OR 1.51 (95% CI 0.80–2.88)	39 (11.1)	OR 1.17 (95% CI 0.71–1.92)
**Physical**					
Body mass index (median, IQR)	25.8 (23.2–29.0)	25.6 (23.5–29.8)	β −0.10 (95% CI −1.06 to 0.86), p = 0.833	26.2 (23.6–29.0)	β −0.51 (95% CI −1.31 to 0.28), p = 0.205
Overweight (N, %)	197 (38.6)	68 (32.2)	OR 1.47 (95% CI 0.99–2.17)	151 (43.1)	OR 0.90 (95% CI 0.66–1.23)
Obesity (N, %)	100 (19.6)	46 (21.8)	OR 0.72 (95% CI 0.46–1.12)	67 (19.1)	OR 0.93 (95% CI 0.63–1.38)
Waist circumference (median, IQR)	89.5 (81.0–98.0)	90.0 (82.0–100.0)	β 0.06 (95% CI −2.82 to 2.94), p = 0.965	91.0 (84.0–99.6)	β −2.00 (95% CI −4.19 to 0.19), p = 0.073
≥threshold (N, %)^d^	234 (45.8)	89 (42.2)	OR 1.13 (95% CI 0.78–1.65)	162 (46.3)	OR 0.97 (95% CI 0.71–1.33)
Leukocytes (10^E^9/L) (median, IQR)	5.90 (5.00–6.05)	5.65 (4.80–6.80)	β 0.10 (95% CI −0.24 to 0.45), p = 0.559	5.90 (5.00–6.90)	β −0.14 (95% CI −0.43 to 0.15), p = 0.330
Neutrophils (10^E^9/L) (median, IQR)	3.22 (2.63–4.01)	3.04 (2.35–3.93)	β 0.12 (95% CI −0.13 to 0.38), p = 0.339	3.16 (2.54–4.01)	β 0.01 (95% CI −0.22 to 0.23), p = 0.965
Lymphocytes (10^E^9/L) (median, IQR)	1.89 (1.56–2.20)	1.93 (1.55–2.27)	β −0.08 (95% CI −0.19 to 0.04), p = 0.185	1.90 (1.54–2.35)	β −0.03 (95% CI −0.13 to 0.07), p = 0.553
Monocytes (10^E^9/L) (median, IQR)	0.46 (0.38–0.55)	0.45 (0.37–0.56)	β −0.000 (95% CI −0.029 to 0.027), p = 0.953	0.48 (0.39–0.57)	β −0.03 (95% CI −0.05 to −0.002), p = 0.031
Eosinophils (10^E^9/L) (median, IQR)	0.17 (0.11–0.24)	0.15 (0.10–0.24)	β 0.000 (95% CI −0.023 to 0.023), p = 1.000	0.16 (0.11–0.23)	β 0.004 (95% CI −0.01 to 0.02), p = 0.607
Basophils (10^E^9/L) (median, IQR)	0.05 (0.03–0.06)	0.04 (0.03–0.06)	β 0.003 (95% CI −0.001 to 0.008), p = 0.117	0.05 (0.03–0.06)	β 0.000 (95% CI −0.004 to 0.004), p = 1.000
Neutrophil-to-lymphocyte ratio (median, IQR)	1.68 (1.35–2.18)	1.51 (1.24–2.11)	β 0.10 (95% CI −0.05 to 0.26), p = 0.181	1.69 (1.35–2.13)	β −0.01 (95% CI −0.14 to 0.11), p = 0.831

SD = standard deviation. IQR = interquartile range. OR = odds ratio. 95% CI = 95% confidence interval. Multinomial, logistic, or quantile regression with a psychological, behavioural, and physical aspects of caregiver strain as dependent variable and being a parental/non-parental (non-)autism-caregiver as independent variable. Model 3 adjusted for age, sex, employment, and educational attainment.

a A higher score in the Long-term Difficulties Inventory resembles more perceived stress.

b A lower score resembles worse perceived health.

c Based on the Short Questionnaire to Assess Health-enhancing physical activity.

d Waist circumference above threshold for metabolic syndrome: ≥88 cm in women, ≥102 cm in men.

As supplementary analyses, Spearman correlation tests were performed to investigate the correlations between the psychological and physical aspects of caregiver strain: see Supplementary Table S2.

### Role of the funding source

The funding source had no role in the study design, collection, analysis and interpretation of data, writing of the report, or the decision to submit this paper for publication. LAN, PJML, MBT, HWH, EFCR, RRJMV, and WAE had access to the data summary and analyses output for review and comment.

## Results

### Basic characteristics

The basic characteristic of the 722 autism-caregivers and 2632 non-autism-caregivers are summarised in Table 1. On average, the autism-caregivers were three years younger than the non-autism-caregivers (50.8 versus 53.8 years old). The autism-caregivers consisted of more females (75%) than the non-autism-caregivers (68%). The majority of the autism-caregivers were parental caregivers (71.9%), while most non-autism-caregivers (61.1%) took care of their mother and/or father (in law). Educational attainment was higher in autism-caregivers than in non-autism-caregivers. Importantly, autistic traits as measured by the AQ-10 sum-scores were not different between autism-caregivers and non-autism-caregivers.

### Psychological aspects of caregiver strain

In Table 2, the psychological, behavioural, and physical aspects of caregiver strain in autism-caregivers compared to non-autism-caregivers are displayed. In this results section, outcomes from model 3 are summarised. Reported chronic stress was higher in autism-caregivers than in non-autism-caregivers (stress ≥ 5: odds ratio (OR) 3.6, 95% confidence interval (CI) 2.60–4.99). Perceived health was not different between autism-caregivers and non-autism-caregivers. Moreover, the prevalence of an anxiety disorder was higher in autism-caregivers than in non-autism-caregivers (12.2% versus 6.9%; OR 1.85, 95% CI 1.37–2.49), as well as the prevalence of a depressive disorder (6.0% versus 2.8%; OR 1.83, 95% CI 1.17–2.86).

### Behavioural aspects of caregiver strain

The prevalence of being physically active during ≥150 min per week (OR 1.12, 95% CI 0.93–1.35), the prevalence of smoking (OR 0.97, 95% CI 0.73–1.31), and alcohol use of more than two glasses per day (OR 0.99, 95% CI 0.73–1.34) were not different between autism-caregivers and non-autism-caregivers.

### Physical aspects of caregiver strain

The mean BMI, mean waist circumference, prevalence of overweight, and prevalence of obesity were not different between autism-caregivers and non-autism-caregivers (see Table 2: p-values ≥ 0.05). With regard to the total leukocyte- and -subtype counts, lymphocyte and monocyte levels were lower in autism-caregivers than in non-autism-caregivers (lymphocytes: β −0.08, p = 0.016; monocytes: β −0.02, p = 0.014).

### Parental autism-caregivers

The basic characteristics of the parental autism-caregivers are summarized in Table 1. The parental autism-caregivers had a lower mean age and consisted of more females than the non-parental autism-caregivers and parental non-autism-caregivers. Furthermore, less parental autism-caregivers had low education attainment and more were employed than the parental non-autism-caregivers. Education and employment were not different between parental and non-parental autism-caregivers.

With respect to the subgroups within autism-caregivers, almost all investigated psychological, behavioural and physical aspects of caregiver strain were not different between the 511 parental autism-caregivers and the 211 non-parental autism-caregivers (column (1) in Table 3: p-values ≥ 0.05). Only the percentage of smokers was higher in parental autism-caregivers than in non-parental autism-caregivers (13.5% versus 7.6%; OR 2.36, 95% CI 1.19–4.70).

When comparing the parental subgroups (column (2) in Table 3), the 511 parental autism-caregivers had higher stress levels (stress ≥ 5: OR 3.10, 95% CI 1.86–5.17), a higher prevalence of anxiety disorders (12.7% versus 6.9%; OR 1.87, 95% CI 1.09–3.25), and higher monocyte levels (β −0.03, p = 0.031) than the 350 parental non-autism-caregivers. Perceived health, the prevalence of a depressive disorder, and the investigated behavioural and other physical aspects of caregiver strain were not different between the parental autism-caregivers and parental non-autism-caregivers (column (2) in Table 3: p-values ≥ 0.05).

## Discussion

The need to gain more insight into multiple psychological, behavioural, and physical aspects of caregiver strain in autism-caregivers is evident because of their increased caregiver strain, since this seems to be associated with adverse health outcomes, and since parental autism-caregiver strain can impact children’s internalizing and externalizing behaviour.^7^^,^^8^ In this study, we observed that being an autism-caregiver is associated with chronic stress, anxiety disorders, depressive disorders, and lower lymphocyte-counts.

Our finding that chronic stress was higher in autism-caregivers than in non-autism-caregivers, as well as in parental autism-caregivers compared to parental non-autism-caregivers, supports the conclusion that autism-caregivers are at greater risk for chronic stress than caregivers for people with other conditions. This is consistent with results from previous studies.^7^^,^^8^ The autism-caregivers in our study reported worse perceived health, but the differences were not statistically significantly different compared to non-autism-caregivers. However, previous research has reported worse perceived health in parents of autistic children than in parents of typically developing children.^6^ Thus, self-reported health should still be assessed in future studies as measure of caregiver strain in autism-caregivers.^5^ In our study, the prevalence of both an anxiety disorder and a depressive disorder were higher in autism-caregivers than in non-autism-caregivers. This finding is partly in line with a recent meta-analysis, which concluded that parents of autistic children have elevated levels of depressive symptoms compared to controls, but levels of anxiety symptoms were not different.^11^ However, the included studies in this meta-analysis used self-reported depression and anxiety symptoms assessed by questionnaire, while in our study, the presence of a depressive or anxiety disorder was assessed with a face-to-face Mini International Neuropsychiatric Interview. Also, another previous study showed that parents of autistic children reported higher levels of depression and anxiety than parents of children without a chronic condition.^24^ Thus, our study results, support the notion that autism-caregivers are at greater risk for both depression and anxiety than non-autism-caregivers, since there are still mixed findings in the field.

It should also be noted that some autistic traits, like sensory over-responsivity, are associated with stress and anxiety.^25^ So, one can debate that the increased risk for psychological adverse outcomes is related to autistic traits in autism-caregivers. However, in our study population, autism-caregivers did not have a higher AQ-10 sum-score than non-autism-caregivers. Thus, our findings do not support the hypothesis that the increased psychological caregiver strain in autism-caregivers is only due to their autistic traits.

It should be noted that the subgroup analysis, comparing parental autism-caregivers with non-parental autism-caregivers, is not a classical subgroup analysis using tests of interaction, but is interpreted qualitatively. Nonetheless, the findings of these subgroup analyses show that the investigated psychological aspects of caregiver strain are not different between these parental and non-parental autism-caregivers.

Based on previous research, we hypothesise that this increased psychological caregiver strain in autism-caregivers could be related to lifelong uncertainties regarding the expected life outcomes of the care-receiver, partly related to the increased risk for psychiatric and somatic comorbities in autistic people.^26^ Secondly, societal stigmas concerning autism might play a role, possibly leading to social isolation of both the autism-caregiver and autistic care-receiver.^7^ These underlying pathways that may contribute to higher psychological caregiver strain in autism-caregivers should be subject of future research.

Our study did not display a difference in physical activity between autism-caregivers and non-autism-caregivers, while previous research showed varying results.^27^^,^^28^ It should be taken into account that caregivers could experience barriers to being physically active, for example because of a lack of time.^28^ In our study, the prevalence of alcohol use of more than two glasses per day in autism-caregivers was not different from non-autism-caregivers. Previous studies observed more alcohol use in parents of autistic children than in controls.^12^^,^^27^ Because of the differences in investigated study populations, more research into alcohol use in autism-caregivers is needed. The prevalence of smoking in autism-caregivers in our study (11.8%) was not different compared to non-autism-caregivers, but the smoking prevalence we observed in autism-caregivers is in line with previous research.^12^ For the subgroup of parental autism-caregivers, smoking cessation interventions should especially be considered, since they smoked more than the non-parental autism-caregivers in our study.

Waist circumference and BMI were not different between autism-caregivers and non-autism-caregivers, nor between parental autism-caregivers and parental non-autism-caregivers. In previous research investigating parental autism-caregivers, BMI was also not different compared to parental non-autism-caregivers.^15^ However, it should be noted that the prevalence of obesity in the autism-caregivers in our study was 20%, which is considerably higher than the prevalence of 14% in the Dutch general population and in line with a recent study into obesity in parental autism-caregivers.^4^ Thus, more research into obesity in (parental and non-parental) autism-caregivers is needed, since previous studies only investigated parental autism-caregivers.

In our study population, lower lymphocyte and monocyte-counts were observed in autism-caregivers than in non-autism-caregivers. In previous research, caregiver stress has been associated with several immunological markers, such as altered levels of interleukin-6, nuclear factor-κB, and T-cells.^29^^,^^30^ In a study including high-stress mothers of autistic children, altered percentages of T-cell subpopulations were detected in these autism-caregivers compared to low-stress mothers of neurotypical children.^30^ Taken together, increased stress levels experienced by autism-caregivers could be related to immunological alterations, including altered leukocyte and monocyte levels, which could hypothetically be explained by the relation between chronic stress and disturbances in the HPA axis. To clarify, there is an association between chronic stress (psychological well-being) and leukocyte-counts in the peripheral blood, mediated through altered gene expression of myeloid cells.^31^ Thus, the immunological alterations in our autism-caregivers endorse that the higher stress levels in autism-caregivers than in non-autism-caregivers might not only self-perceived, but could also be reflected in biological measures linked to the HPA axis.

The main strength of this study is the large prospective study cohort, in which we investigated caregiver strain including biomarkers and other clinical measurements, and not merely relying on self-reported health. Accordingly, we assessed stress levels using both self-report questionnaires and a laboratory approach including immunological blood markers. To our knowledge, the overview of relevant psychological, behavioural, and biological aspects of caregiver strain in a large caregiver-population that we offer in this study is a significant contribution to previous research in the field.

We were limited, however, in the way and time the various measurements were assessed in the Lifelines cohort (Fig. 1). The caregiver questionnaire (AUTQ) was submitted in 2019, while the psychological, behavioural, and physical aspects were assessed in the time frame of 2014–2017, leading to a time gap of 2–5 years. However, we only included participants who reported to be caregiver since 2014 or earlier, to ensure the caregiving exposure began prior to the measurement of aspects of caregiver strain. Due to the study design, it is important to note that direct causality between being an autism-caregiver and the psychological, behavioural, and physical outcomes cannot be proved. Thus, for clinical practice, our study results offer insights into associations between autism-caregiving and psychological, behavioural, and physical aspects of caregiver strain, but to unravel underlying causal pathways, further research is needed.

The worse psychological aspects of caregiver strain and altered immune balance we found in autism-caregivers compared with non-autism-caregivers imply that autism-caregivers are at higher risk for adverse chronic health outcomes. Implementation of (preventive) interventions focusing on improvement of autism-caregivers’ health should be the objective of future studies. It is important that not only parents of autistic children (parental autism-caregivers) are being included in future preventive caregiver strain interventions, but also non-parental autism-caregivers, since the parental and non-parental autism-caregivers in our study experienced similar psychological caregiver strain. Moreover, future research including older caregivers who are giving care to their autistic child throughout their child’s lifetime, could be valuable in order to investigate the long-term associations between being an autism-caregiver and adverse health outcomes. This is especially relevant for autism-caregivers, since autism is a life-long condition, which often requires life-long informal care.

Autism-caregivers are characterised by higher levels of psychological stress and a higher prevalence of anxiety and depressive disorders compared to non-autism-caregivers. In addition, autism-caregiving is related to an altered lymphocyte balance, which could hypothetically be related to increased stress. The results of our subgroup analyses regarding parental versus non-parental autism-caregivers show that these subgroups are rather similar regarding caregiver strain. Taken together, reduction of their stress, anxiety and depression in autism-caregivers is needed. Interventions aiming at improvement of autism-caregivers’ health and reduction of their caregiver strain could be focus of future research, and eventually might contribute to reduction of their increased morbidity and mortality risk.

## Contributors

Conceptualization of this paper was done by EBW, SEL, LAN, MBT, RJMV, and WAE. Literature search was conducted by EBW. EBW and SEL had access to the direct data and verified the underlying data. LAN, PJML, MBT, HWH, EFCR, RRJMV, and WAE had access to the data summary and analyses output for review and comment. The statistical analyses were performed by EBW and SEL, with supervision of MBT and WAE. The original draft was written by EBW with review and editing by EBW, SEL, LAN, PJML, MBT, HWH, EFCR, RRJMV, and WAE.

## Data sharing statement

All data collected for the study, including individual (pseudonymized) participant data and a data dictionary defining each field in the set, are available via the Lifelines Research Office (https://www.lifelines.nl/researcher/how-to-apply/). Access to this dataset and other available data and samples from the Lifelines cohort can be requested by scientists working in the field of “healthy ageing”. Access will be granted after evaluation of an application form describing the research proposal (including a data selection) and a signed Data and Material Transfer Agreement. Data will be released in a secure environment.

## Declaration of interests

The authors declare not having any conflict of interest.

## Appendix ASupplementary data

Supplementary Tables S1 and S2

STROBE checklist
