# Supplementary material for: Psychological, behavioural, and physical aspects of caregiver strain in autism-caregivers: a cohort study
Source: eClinicalMedicine. 2023 Sep 20;64:102211. doi: 10.1016/j.eclinm.2023.102211 (PMC10520302; doi:10.1016/j.eclinm.2023.102211)
Supplement: Supplementary Tables S1 and S2 [file mmc1.pdf]

Supplementary material

Supplementary table 1. Missing data in covariates and outcome measures

	Autism-caregivers n=722	Non-autism-caregivers n=2632
**Covariates**		
Age, missing (N, %)	0 (0)	0 (0)
Female sex, missing (N, %)	0 (0)	0 (0)
Educational attainment, missing (N, %)	147 (20·4)	441 (16·8)
Employment, missing (N, %)	72 (10·0)	174 (6·6)
**Outcome measures**		
Stress, missing (N, %)	73 (10·1)	180 (6·8)
Perceived health, missing (N, %)	71 (9·8)	176 (6·7)
Anxiety disorder, missing (N, %)	159 (22·0)	580 (22·0)
Depressive disorder, missing (N, %)	159 (22·0)	580 (22·0)
Physical activity, missing (N, %)	75 (10·4)	184 (7·0)
Smoking, missing (N, %)	118 (16·3)	306 (11·6)
Alcohol use, missing (N, %)	332 (46·0)	1065 (40·5)
Body mass index, missing (N, %)	0 (0)	0 (0)
Overweight, missing (N, %)	0 (0)	0 (0)
Obesity, missing (N, %)	0 (0)	0 (0)
Waist circumference, missing (N, %)	0 (0)	0 (0)
≥ threshold, missing (N, %)	0 (0)	0 (0)
Leukocytes, missing (N, %)	22 (3·0)	88 (3·3)
Neutrophils, missing (N, %)	22 (3·0)	88 (3·3)
Lymphocytes, missing (N, %)	22 (3·0)	88 (3·3)
Monocytes, missing (N, %)	22 (3·0)	88 (3·3)
Eosinophils, missing (N, %)	22 (3·0)	88 (3·3)
Basophils, missing (N, %)	22 (3·0)	88 (3·3)
Neutrophil-to-lymphocyte ratio, missing (N, %)	22 (3·0)	88 (3·3)

Supplementary table 2. Spearman correlations between psychological and physical measures in total study population

	Stress	Perceived health	Anxiety disorder	Depressive disorder
Body mass index	CC 0·005, p=0·780	CC -0·185, p<0·001 *	CC 0·007, p=0·711	CC 0·032, p=0·105
Waist circumference	CC -0·042, p=0.018 *	CC -0·178, p<0·001 *	CC 0·007, p=0·703	CC 0·025, p=0·202
Leukocytes	CC 0·060, p=0·001 *	CC -0·116, p<0·001 *	CC 0·024, p=0·233	CC 0·026, p=0·183
Neutrophils	CC 0·084, p<0·001 *	CC -0·107, p<0·001 *	CC 0·036, p=0·066	CC 0·021, p=0·297
Lymphocytes	CC 0·007, p=0·684	CC -0·074, p<0·001 *	CC -0·015, p=0·434	CC 0·020, p=0·316
Monocytes	CC -0·030, p=0·100	CC -0·056, p=0·002 *	CC -0·011, p=0·564	CC 0·023, p=0·237
Eosinophils	CC -0·009, p=0·638	CC -0·056, p=0·002 *	CC 0·005, p=0·794	CC 0·010, p=0·602
Basophils	CC 0·019, p=0·295	CC -0·027, p=0·144	CC 0·004, p=0·824	CC 0·002, p=0·929
Neutrophil-to-lymphocyte ratio	CC 0·066, p<0·001 *	CC -0·032, p=0·078	CC 0·043, p=0·030 *	CC 0·001, p=0·979

CC = correlation coefficient

Statistically significant correlation coefficients are marked: *.
