# Supplementary material for: Psychological, behavioural, and physical aspects of caregiver strain in autism-caregivers: a cohort study
Source: eClinicalMedicine. 2023 Sep 20;64:102211. doi: 10.1016/j.eclinm.2023.102211 (PMC10520302; doi:10.1016/j.eclinm.2023.102211)
Supplement: STROBE checklist [file mmc2.docx]

STROBE Statement—checklist of items that should be included in reports of observational studies

|  | Item No. | Recommendation | Page  No. | Relevant text from manuscript |
| --- | --- | --- | --- | --- |
| **Title and abstract** | 1 | (*a*) Indicate the study’s design with a commonly used term in the title or the abstract | 1-2 | “Lifelines Cohort” |
|  |  | (*b*) Provide in the abstract an informative and balanced summary of what was done and what was found | 2-3 | Background, Methods, Findings, Interpretation |
| Introduction | | | |  |
| Background/rationale | 2 | Explain the scientific background and rationale for the investigation being reported | 4-5 | Introduction of topic of caregiver-strain in autism-caregivers and gap in knowledge for psychological, behavioral and physical factors, leading to rationale of the study |
| Objectives | 3 | State specific objectives, including any prespecified hypotheses | 5 | “The first aim of this study is to compare the above-mentioned psychological, behavioral, and physical aspects of caregiver-strain in autism-caregivers and non-autism-caregivers. Moreover, we hypothesize that autism-caregiving is associated with higher caregiver-strain than non-autism-caregiving, irrespective of being a parent. To investigate this second hypothesis, we also aimed to compare the above-mentioned psychological, behavioral, and physical aspects of caregiver-strain between parental autism-caregivers and non-parental autism-caregivers, and between parental autism-caregivers and parental non-autism-caregivers.” |
| Methods | | | |  |
| Study design | 4 | Present key elements of study design early in the paper | 7 & Figure 1 | Reference to Figure 1 for the study design |
| Setting | 5 | Describe the setting, locations, and relevant dates, including periods of recruitment, exposure, follow-up, and data collection | 7 | Study population description and reference to Figure 1 |
| Participants | 6 | (*a*) *Cohort study*—Give the eligibility criteria, and the sources and methods of selection of participants. Describe methods of follow-up  *Case-control study*—Give the eligibility criteria, and the sources and methods of case ascertainment and control selection. Give the rationale for the choice of cases and controls  *Cross-sectional study*—Give the eligibility criteria, and the sources and methods of selection of participants | 7 & Figure 2 | “We included 3354 participants who reported being a caregiver since 2014 (the start of the second Lifelines assessment) or earlier, aged 18 years or older during the physical visit of the second Lifelines assessment (Figure 2). In this way, the caregiving began prior to the measurement of caregiver-strain. Informal caregiving was defined as giving unpaid care to someone close with long-term limitations and/or health problems. ‘Someone close’ was described as a partner, a relative (including a child), a friend, or someone else close to you. Volunteer work was not included in our definition of caregiving. Parental caregivers were adults who gave care to their son or daughter (in law). Non-parental caregivers were adults who gave care to their partner, mother (in law), father (in law), brother, sister, friend, acquaintance, or neighbor.” |
|  |  | (*b*) *Cohort study*—For matched studies, give matching criteria and number of exposed and unexposed  *Case-control study*—For matched studies, give matching criteria and the number of controls per case |  |  |
| Variables | 7 | Clearly define all outcomes, exposures, predictors, potential confounders, and effect modifiers. Give diagnostic criteria, if applicable | 7-10 | Definitions of caregivers, descriptions of all measures and covariates |
| Data sources/ measurement | 8* | For each variable of interest, give sources of data and details of methods of assessment (measurement). Describe comparability of assessment methods if there is more than one group | 7-10 | Definitions of caregivers, descriptions of all measures and covariates: assessed with questionnaires or physical measurements |
| Bias | 9 | Describe any efforts to address potential sources of bias | 10 | “In order to evaluate potential selection bias, we executed a non-response analysis to map characteristics of the 71,428 Lifelines participants that did receive the AUTQ, but did not submit this questionnaire (and could therefore not be included in our study). This non-response analysis showed that these 71,428 non-eligible Lifelines participants were younger in age and consisted of more men compared to the 37,924 participants who did submit this AUTQ questionnaire.” |
| Study size | 10 | Explain how the study size was arrived at | 7-8 and Figure 2 | Study population inclusion in text and summarized in Figure 2 |

Continued on next page

| Quantitative variables | 11 | Explain how quantitative variables were handled in the analyses. If applicable, describe which groupings were chosen and why | 10 | All variables were quantitative and analyses were described in the Statistical analyses paragraph. |
| --- | --- | --- | --- | --- |
| Statistical methods | 12 | (*a*) Describe all statistical methods, including those used to control for confounding | 10 | Statistical analyses paragraph |
|  |  | (*b*) Describe any methods used to examine subgroups and interactions | 10 | Subgroup analyses were the same as for the main group, described in the Statistical analyses paragraph. |
|  |  | (*c*) Explain how missing data were addressed | 10-11 | Missing data from covariates and outcome measures were shown in Supplementary Table 1. “Because of missing data in the covariates of employment and educational attainment (see all missing data in Supplementary Table 1), we executed step-by-step analyses: model 1 was only adjusted for age and sex; in model 2, adjustment for employment was added; in model 3, adjustment for educational attainment was added.” |
|  |  | (*d*) *Cohort study*—If applicable, explain how loss to follow-up was addressed  *Case-control study*—If applicable, explain how matching of cases and controls was addressed  *Cross-sectional study*—If applicable, describe analytical methods taking account of sampling strategy | Figure 2 | Figure 2 |
|  |  | (*e*) Describe any sensitivity analyses | NA |  |
| Results | | | | |
| Participants | 13* | (a) Report numbers of individuals at each stage of study—eg numbers potentially eligible, examined for eligibility, confirmed eligible, included in the study, completing follow-up, and analysed | 8 and Figure 2 | “Of the 3354 included caregivers (Figure 2), 722 were autism-caregiver and 2632 were non-autism-caregiver. These autism-caregivers consisted of 511 parental autism-caregivers (71.9%). In the group of non-autism-caregivers, there were 350 parental non-autism-caregivers (13.3%).” |
|  |  | (b) Give reasons for non-participation at each stage | Figure 2 | Figure 2 |
|  |  | (c) Consider use of a flow diagram | Figure 2 | Flow diagram in Figure 2 |
| Descriptive data | 14* | (a) Give characteristics of study participants (eg demographic, clinical, social) and information on exposures and potential confounders | 12 | “The basic characteristic of the 722 autism-caregivers and 2632 non-autism-caregivers are summarized in Table 1. On average, the autism-caregivers were three years younger than the non-autism-caregivers (50.8 versus 53.8 years old). The autism-caregivers consisted of more females (75%) than the non-autism-caregivers (68%). The majority of the autism-caregivers were parental caregivers (71.9%), while most non-autism-caregivers (61.1%) took care of their mother and/or father (in law). Educational attainment was higher in autism-caregivers than in non-autism-caregivers. Importantly, autistic traits as measured by the short version of the Autism Spectrum Quotient (AQ-10) sum-scores were not different between autism-caregivers and non-autism-caregivers.” |
|  |  | (b) Indicate number of participants with missing data for each variable of interest | Supplementary tables | Tables in Supplementary material regarding missing data for each variable. |
|  |  | (c) *Cohort study*—Summarise follow-up time (eg, average and total amount) | 6 and Figure 1 | “Baseline assessment was performed from 2007 until 2013, and the second assessment took place between 2014 and 2017 (Figure 1). In 2019, 109,352 participants received an autism- and caregiver-questionnaire (AUTQ).” |
| Outcome data | 15* | *Cohort study*—Report numbers of outcome events or summary measures over time |  |  |
|  |  | *Case-control study—*Report numbers in each exposure category, or summary measures of exposure | 12-13 and Table 2 and 3 | Paragraphs regarding Psychological, behavioral and physical aspects of caregiver-strain |
|  |  | *Cross-sectional study—*Report numbers of outcome events or summary measures |  |  |
| Main results | 16 | (*a*) Give unadjusted estimates and, if applicable, confounder-adjusted estimates and their precision (eg, 95% confidence interval). Make clear which confounders were adjusted for and why they were included | 12-13 and Table 2 and 3 | Paragraphs regarding Psychological, behavioral and physical aspects of caregiver-strain and corresponding Tables 2 and 3 |
|  |  | (*b*) Report category boundaries when continuous variables were categorized | 8 | Measures |
|  |  | (*c*) If relevant, consider translating estimates of relative risk into absolute risk for a meaningful time period | NA |  |

Continued on next page

| Other analyses | 17 | Report other analyses done—eg analyses of subgroups and interactions, and sensitivity analyses | 12-13 and Table 2 and 3 | Paragraphs regarding Psychological, behavioral and physical aspects of caregiver-strain and corresponding Tables 2 and 3 |
| --- | --- | --- | --- | --- |
| Discussion | | | | |
| Key results | 18 | Summarise key results with reference to study objectives | 14-19 | Whole discussion and conclusion |
| Limitations | 19 | Discuss limitations of the study, taking into account sources of potential bias or imprecision. Discuss both direction and magnitude of any potential bias | 17 | “We were limited, however, in the way and time the various measurements were assessed in the Lifelines cohort (Figure 1). The caregiver questionnaire (AUTQ) was submitted in 2019, while the psychological, behavioral, and physical aspects were assessed in the time frame of 2014-2017, leading to a time gap of 2-5 years. However, we only included participants who reported to be caregiver since 2014 or earlier, to ensure the caregiving exposure began prior to the measurement of aspects of caregiver-strain. Due to the study design, it is important to note that direct causality between being a caregiver and the psychological, behavioral, and physical outcomes cannot be proved.” |
| Interpretation | 20 | Give a cautious overall interpretation of results considering objectives, limitations, multiplicity of analyses, results from similar studies, and other relevant evidence | 18 | “The worse psychological aspects of caregiver-strain and altered immune balance we found in autism-caregivers compared with non-autism-caregivers imply that autism-caregivers are at higher risk for adverse chronic health outcomes. Implementation of (preventive) interventions focusing on improvement of autism-caregivers’ health should be the objective of future studies. It is important that not only parents of autistic children (parental autism-caregivers) are being included in future preventive caregiver-strain interventions, but also non-parental autism-caregivers, since the parental and non-parental autism-caregivers in our study experienced equal psychological caregiver-strain. Moreover, future research including older caregivers who are giving care to their autistic child throughout their child’s lifetime, could be valuable in order to investigate the long-term associations between being an autism-caregiver and adverse health outcomes. This is especially relevant for autism-caregivers, since autism is a life-long condition, which often requires life-long informal care.” |
| Generalisability | 21 | Discuss the generalisability (external validity) of the study results | 17 | “The Lifelines Cohort is representative for the general population in the Northern Netherlands, as participants were recruited through their general practitioners.” |
| Other information | |  | | |
| Funding | 22 | Give the source of funding and the role of the funders for the present study and, if applicable, for the original study on which the present article is based | 19 | “The Lifelines initiative has been made possible by subsidy from the Dutch Ministry of Health, Welfare and Sport, the Dutch Ministry of Economic Affairs, the University Medical Center Groningen, and the Provinces in the North of the Netherlands (Drenthe, Friesland, Groningen).” |

*Give information separately for cases and controls in case-control studies and, if applicable, for exposed and unexposed groups in cohort and cross-sectional studies.

**Note:** An Explanation and Elaboration article discusses each checklist item and gives methodological background and published examples of transparent reporting. The STROBE checklist is best used in conjunction with this article (freely available on the Web sites of PLoS Medicine at http://www.plosmedicine.org/, Annals of Internal Medicine at http://www.annals.org/, and Epidemiology at http://www.epidem.com/). Information on the STROBE Initiative is available at www.strobe-statement.org.
